# Beyond depression symptoms: the default mode network as a predictor of antidepressant response

**DOI:** 10.1038/s44184-025-00182-2

**Published:** 2026-01-16

**Authors:** Kaizhong Zheng, Liangjun Chen, Huaning Wang, DIRECT consortium, Li-Ping Cao, Guan-Mao Chen, Jian-Shan Chen, Tao Chen, Tao-Lin Chen, Yu-Qi Cheng, Zhao-Song Chu, Shi-Xian Cui, Xi-Long Cui, Zhao-Yu Deng, Qi-Yong Gong, Wen-Bin Guo, Can-Can He, Zheng-Jia-Yi Hu, Qian Huang, Xin-Lei Ji, Feng-Nan Jia, Li Kuang, Bao-Juan Li, Feng Li, Hui-Xian Li, Tao Li, Tao Lian, Yi-Fan Liao, Xiao-Yun Liu, Yan-Song Liu, Zhe-Ning Liu, Yi-Cheng Long, Jian-Ping Lu, Jiang Qiu, Xiao-Xiao Shan, Tian-Mei Si, Peng-Feng Sun, Chuan-Yue Wang, Hua-Ning Wang, Xiang Wang, Ying Wang, Yu-Wei Wang, Xiao-Ping Wu, Xin-Ran Wu, Yan-Kun Wu, Chun-Ming Xie, Guang-Rong Xie, Peng Xie, Xiu-Feng Xu, Zhen-Peng Xue, Hong Yang, Hua Yu, Min-Lan Yuan, Yong-Gui Yuan, Ai-Xia Zhang, Jing-Ping Zhao, Ke-Rang Zhang, Wei Zhang, Zi-Jing Zhang, Chao-Gan Yan, Baojuan Li, Badong Chen

**Affiliations:** 1 National Key Laboratory of Human-Machine Hybrid Augmented Intelligence, National Engineering Research Center for Visual Information and Applications, and Institute of Artificial Intelligence and Robotics, Xi’an Jiaotong University, Xi’an, Shaanxi China; 2 Xijing Hospital, Fourth Military Medical University, Xi’an, Shaanxi China; 3 School of Biomedical Engineering, Fourth Military Medical University, Xi’an, Shaanxi China; 4 Affiliated Brain Hospital of Guangzhou Medical University, Guangzhou, China; 5 The First Affiliated Hospital of Jinan University, Guangzhou, China; 6 Department of Radiology, The First Affiliated Hospital, College of Medicine, Zhejiang University, Hangzhou, China; 7 Huaxi Magnetic Resonance Research Center, Department of Radiology, West China Hospital of Sichuan University, Chengdu, China; 8 Research Unit of Psychoradiology, Chinese Academy of Medical Sciences, Chengdu, China; 9 Department of Psychiatry, First Affiliated Hospital of Kunming Medical University, Kunming, China; 10 Chinese Academy of Sciences Key Laboratory of Behavioral Science, Institute of Psychology, Chinese Academy of Sciences, Beijing, China; 11 Sino-Danish College, University of Chinese Academy of Sciences, Beijing, China; 12 Sino-Danish Center for Education and Research, Graduate University of Chinese Academy of Sciences, Beijing, China; 13 Department of Psychiatry and National Clinical Research Center for Mental Disorders, The Second Xiangya Hospital of Central South University, Changsha, China; 14 Department of Neurology, Affiliated ZhongDa Hospital of Southeast University, Nanjing, China; 15 Department of Psychiatry, The First Affiliated Hospital of Chongqing Medical University, Chongqing, China; 16 Department of Clinical Psychology, Suzhou Psychiatric Hospital, The Affiliated Guangji Hospital of Soochow University, Suzhou, China; 17 Beijing Anding Hospital, Capital Medical University, Beijing, China; 18 Affiliated Mental Health Center and Hangzhou Seventh People’s Hospital, Zhejiang University School of Medicine, Hangzhou, China; 19 Mental Health Center and Psychiatric Laboratory, West China Hospital of Sichuan University, Chengdu, China; 20 Department of Psychosomatics and Psychiatry, Zhongda Hospital, School of Medicine, Southeast University, Nanjing, China; 21 Shenzhen Kangning Hospital, Shenzhen, China; 22 Faculty of Psychology, Southwest University, Chongqing, China; 23 National Clinical Research Center for Mental Disorders (Peking University Sixth Hospital) and Key Laboratory of Mental Health, Ministry of Health (Peking University), Beijing, China; 24 Xi’an Central Hospital, Xi’an, China; 25 Institute of Neuroscience, Chongqing Medical University, Chongqing, China; 26 Chongqing Key Laboratory of Neurobiology, Chongqing, China; 27 Department of Neurology, The First Affiliated Hospital of Chongqing Medical University, Chongqing, China; 28 West China Hospital of Sichuan University, Chengdu, Sichuan China; 29 First Hospital of Shanxi Medical University, Taiyuan, China

**Keywords:** Biomarkers, Diseases, Neurology, Neuroscience, Psychology, Psychology

## Abstract

Antidepressant efficacy for major depressive disorder (MDD) remains limited, with the neural mechanisms underlying treatment response poorly understood. The default mode network (DMN), particularly the connectivity between the medial prefrontal cortex (mPFC) and posterior cingulate cortex (PCC), has been implicated in MDD pathophysiology and may be linked to treatment outcomes. However, its potential as a biomarker for antidepressant response has not been validated. Here, we investigate the relationship between DMN connectivity and antidepressant treatment response in MDD. Resting-state fMRI data from four large MDD cohorts (*n* = 4271) were analyzed using Granger causality to examine directional effective connectivity (EC) within the DMN. Linear mixed-effects models compared EC between recurrent MDD patients, first-episode drug-naïve patients, and healthy controls. We also examined associations between EC, medication use, illness duration, depressive symptoms, and treatment outcomes. Additionally, Support Vector Machine (SVM) classifiers and support vector regression (SVR) were trained using EC from mPFC to PCC to predict treatment response. Our results revealed that recurrent MDD patients exhibited significantly reduced EC from mPFC to PCC compared to healthy controls and first-episode patients, with this reduction correlating with antidepressant medication use and illness duration. Importantly, DMN connectivity was associated with treatment improvement rather than core depressive symptoms, including suicide, anhedonia, or emotional blunting. Crucially, EC from mPFC to PCC predicted antidepressant treatment response, and SVM classifiers demonstrated high predictive accuracy for therapeutic outcomes. In conclusion, reduced EC from mPFC to PCC may serve as a biomarker for antidepressant treatment response in MDD, offering insights into MDD neurobiology and supporting the clinical potential of DMN connectivity measures for guiding treatment decisions. The SAINT, Xijing_QG, and Xijing_KG datasets were approved by the Ethics Committee of the First Affiliated Hospital, Fourth Military Medical University (approval numbers: KY20202066-F-1, XJLL-KY20222111, and KY20222165-F-1, respectively) and registered with clinicaltrials.gov (identifiers: NCT 04653337, NCT 05577481, and NCT 05544071, respectively).

## Introduction

Major depressive disorder (MDD) is a devastating psychiatric affliction, ranking as the second leading cause of global disability, boasting a point prevalence that surpasses 4%^1^. Despite the widespread use of antidepressant medication^2^, its therapeutic efficacy is not entirely satisfactory with only a modest superiority over placebo (Cohen’s d of ~0.3)^3^. Moreover, ~50% of patients with MDD could be treatment-resistant depression^4^. However, the neural mechanisms that underlie antidepressant treatment remain elusive, and the lack of reliable biomarkers poses a hindrance to the effective prediction of treatment responses.

Default mode network (DMN) is thought to play a pivotal role in the pathophysiology of MDD. A plethora of evidence suggests a correlation between the Default Mode Network and the primary clinical symptom of rumination in major depression^5,6^. A recent study revealed a decrease in functional connectivity (FC) within the DMN when comparing 848 patients with MDD to 794 normal controls (NCs)^7^. Importantly, this effect was only statistically significant in individuals with recurrent MDD, as opposed to those with first-episode drug-naïve (FEDN) MDD. Additionally, fMRI-characterization of DMN could serve as a complement to the existing symptom-based diagnoses for MDD. By incorporating increased functional connectivity in the DMN, a support vector machine (SVM) classifier was able to effectively distinguish between patients with MDD and NCs, achieving an AUC of 90%^8^. Moreover, abnormal dynamic functional network connectivity estimated from the DMN has been shown to be predictive of symptom severity in MDD^9^.

Accumulating evidence shows that DMN might be closely associated with treatment response for MDD. The identified hyperconnectivity within the DMN served as a distinguishing factor between patients diagnosed with treatment-resistant MDD and those exhibiting treatment sensitivity^10^. Moreover, research findings have suggested that the observed hyperconnectivity within the DMN and the connections between the DMN and the executive control network (ECN) offered potential as indicators of successful treatment^11^. However, the validation of the DMN as a biomarker for antidepressant treatment is yet to be established.

The medial prefrontal–limbic circuitry is thought to be a key neural pathway underlying the therapeutic effects of antidepressant treatment^12–14^. Previous studies have demonstrated that antidepressants could modulate emotional processing, exerting neural effects within the prefrontal and limbic circuitry^12^. In addition, both patients and healthy controls exhibited increased activity in the medial prefrontal and core limbic regions in response to positive emotions, while these regions showed decreased activity to negative emotions following repeated administration of antidepressants^13^. The therapeutic effects of antidepressant treatment are mediated through “top-down” influences from the prefrontal cortex to the limbic regions^15^. Thus, we further focused on two key regions of the DMN, the medial prefrontal cortex (mPFC) and the posterior cingulate cortex (PCC)—one located in the prefrontal cortex and the other within the limbic system—to investigate the relationship between information flow from the mPFC to the PCC and antidepressant treatment, and to evaluate its potential as a biomarker.

**Fig. 1:**
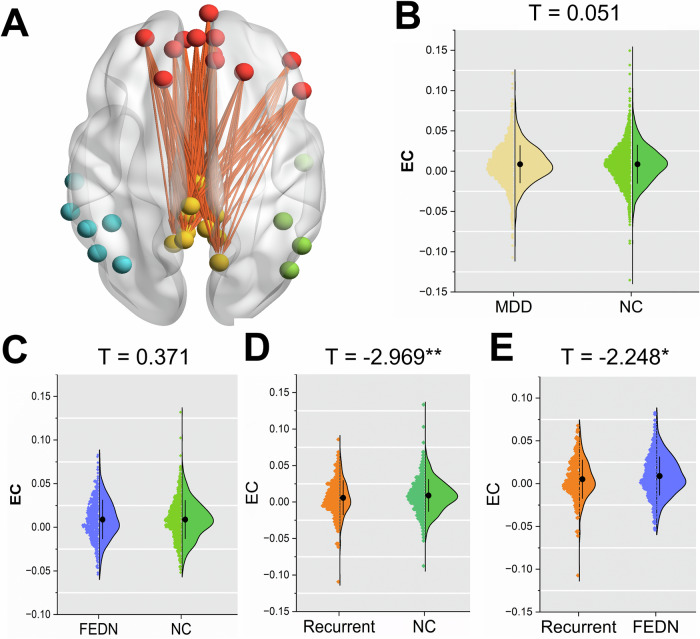
Reduced EC from mPFC to PCC within DMN in patients with recurrent MDD. The average EC from mPFC to PCC was computed across 132 connections, as illustrated in (**A**). Violin plots depict the distribution of mean EC from mPFC to PCC differences among various groups: **B** MDD vs. NC; **C** First Episode Drug Naïve (FEDN) MDD vs. NC; **D** Recurrent MDD vs. NC; and **E** FEDN MDD vs. Recurrent MDD. It is noteworthy that, for each comparison, only sites with a sample size larger than 10 in each group were considered. The *t* values represent the statistics for these comparisons in Linear Mixed-Effects Model (LMM) analyses. *p < 0.05; **p < 0.01.

**Fig. 2:**
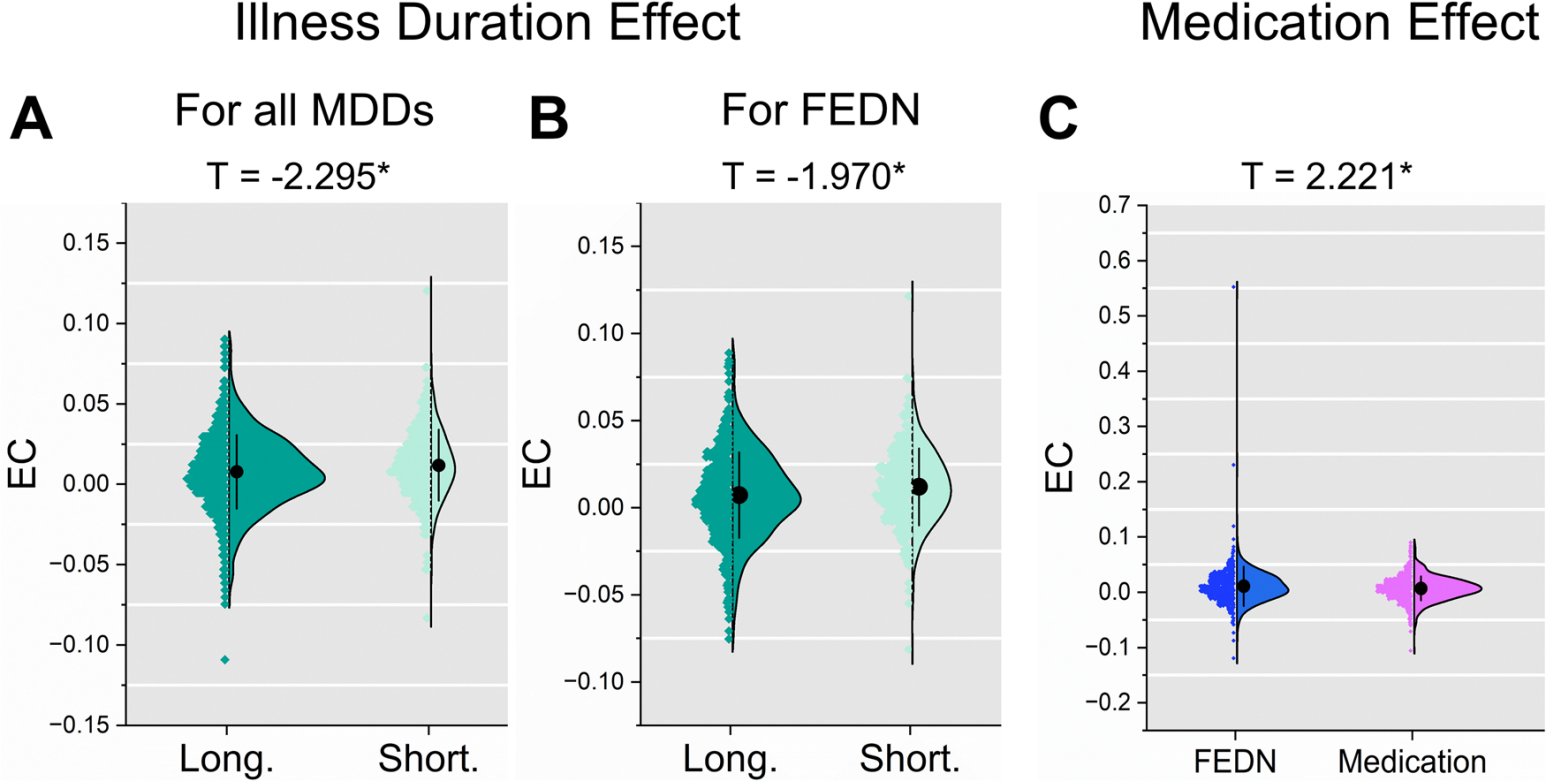
The effects of illness duration and medication status on reduced EC from mPFC to PCC within DMN in MDD patients. The violin figures show the distribution of mean EC from mPFC to PCC within DMN for FEDN MDD patients with long vs. short illness duration (**A**), for all MDD patients with long vs. short illness duration (**B**), and for FEDN MDD patients with vs. without medication usage (**C**). The *t* values represent the statistics for these comparisons in Linear Mixed-Effects Model (LMM) analyses. *p < 0.05.

**Fig. 3:**
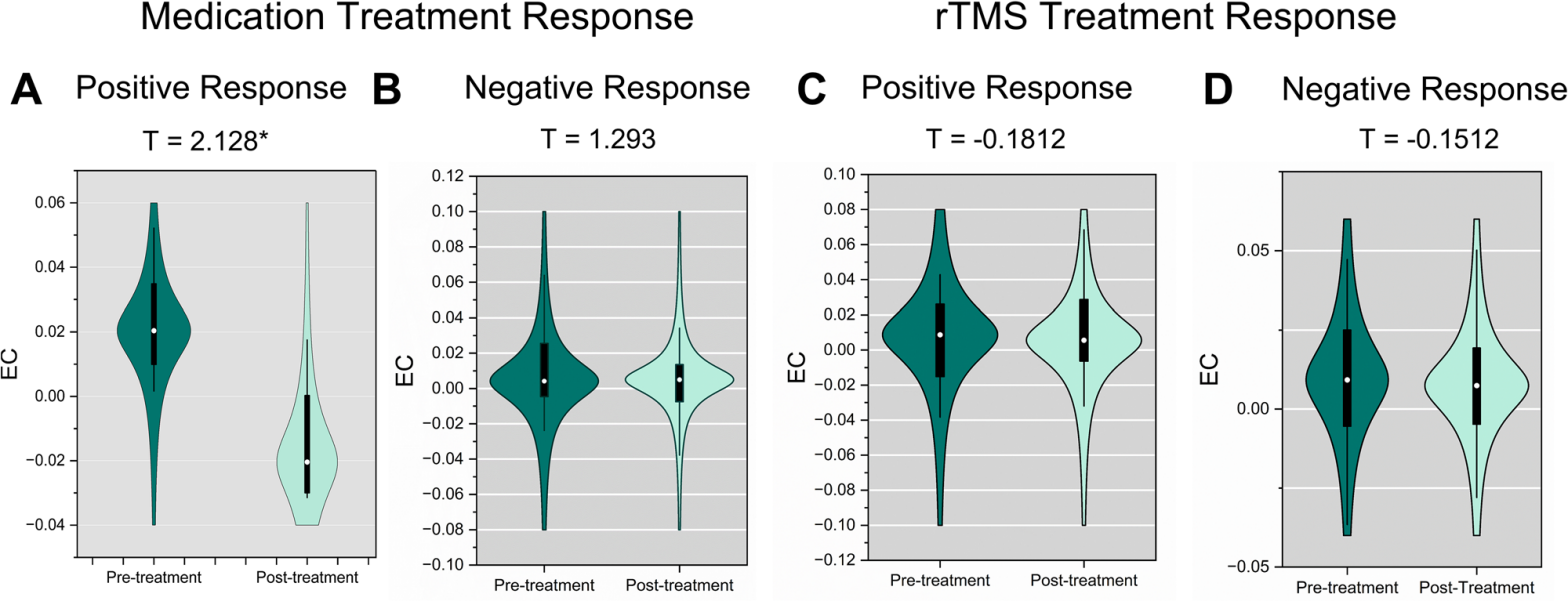
The effects of antidepressant treatment (medication and rTMS) on EC from mPFC to PCC within DMN in MDD patients. The violin figures show the distribution of mean EC from mPFC to PCC within DMN for MDD patients with a positive medication response: pre- versus post-treatment (**A**), for MDD patients with a negative medication response: pre- versus post-treatment (**B**), for MDD patients with a positive rTMS response: pre- versus post-treatment (**C**), and for MDD patients with a negative rTMS response: pre- versus post-treatment (**D**). Here, responders are identified as individuals exhibiting a reduction of 50% or greater on the HAMD-17 scale following the specific antidepressant treatment (positive response), while non-responders are those who do not meet this threshold (negative response). *p < 0.05.

**Fig. 4:**
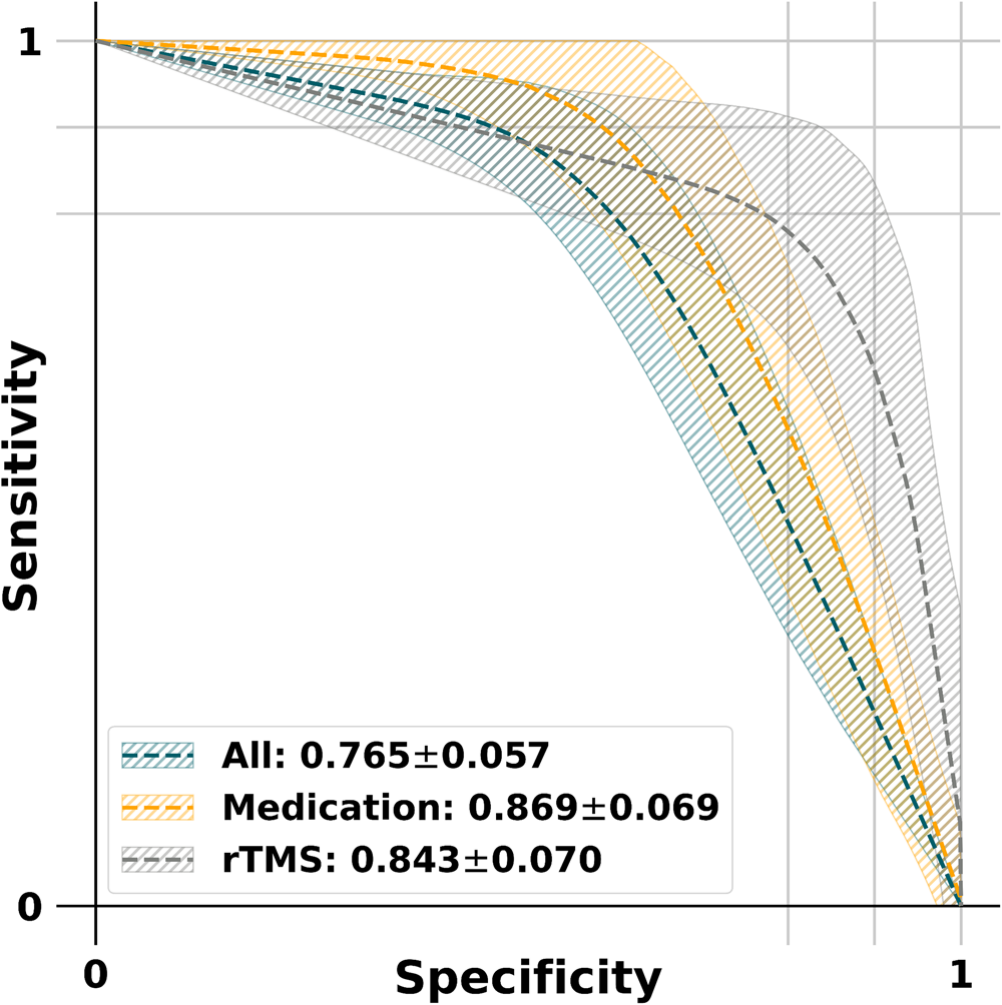
Prediction of outcome specific to antidepressant treatment using EC specifically from the medial prefrontal cortex (mPFC) to the posterior cingulate cortex (PCC). Sensitivity-specificity curves based on model predictions for predicting the response to all treatment (213 responders versus 102 non-responders), medication treatment (173 responders versus 56 non-responders) and rTMS treatment (40 responders versus 46 non-responders). The calculation of area under the curve (AUC) values was performed for each sensitivity-specificity curve.

**Fig. 5:**
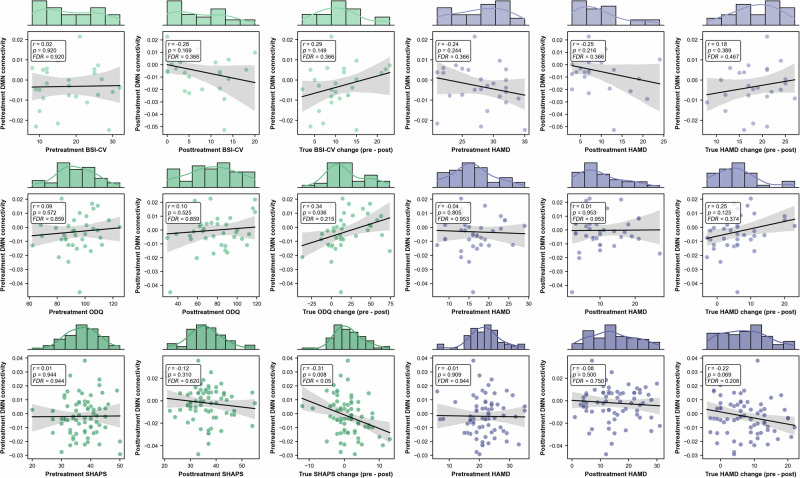
Association of EC from mPFC to PCC with BSI-CV, HAMD, ODQ and SHAPS. Rows 1–3 correspond to the SAINT, Xijing_QG, and Xijing_KG datasets, respectively.

**Fig. 6:**
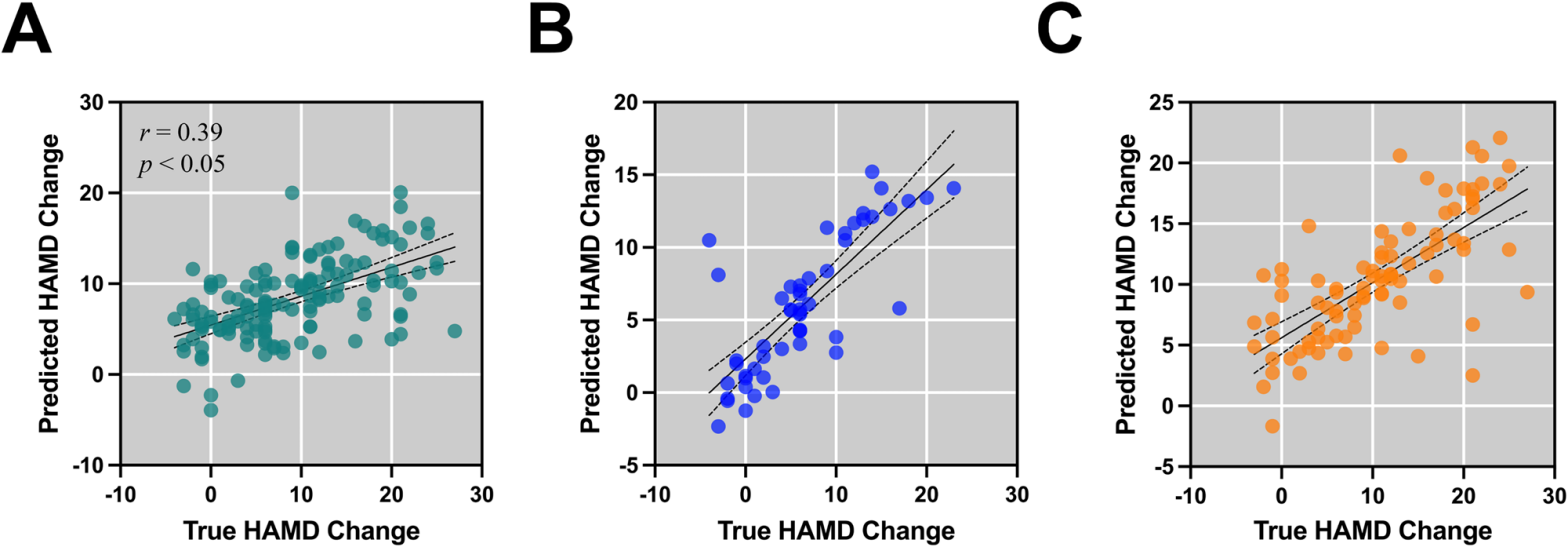
Predicting changes in HAMD scores following different treatment modalities using EC from mPFC to PCC. **A** All treatment, **B** medication treatment, **C** rTMS treatment.

**Fig. 7:**
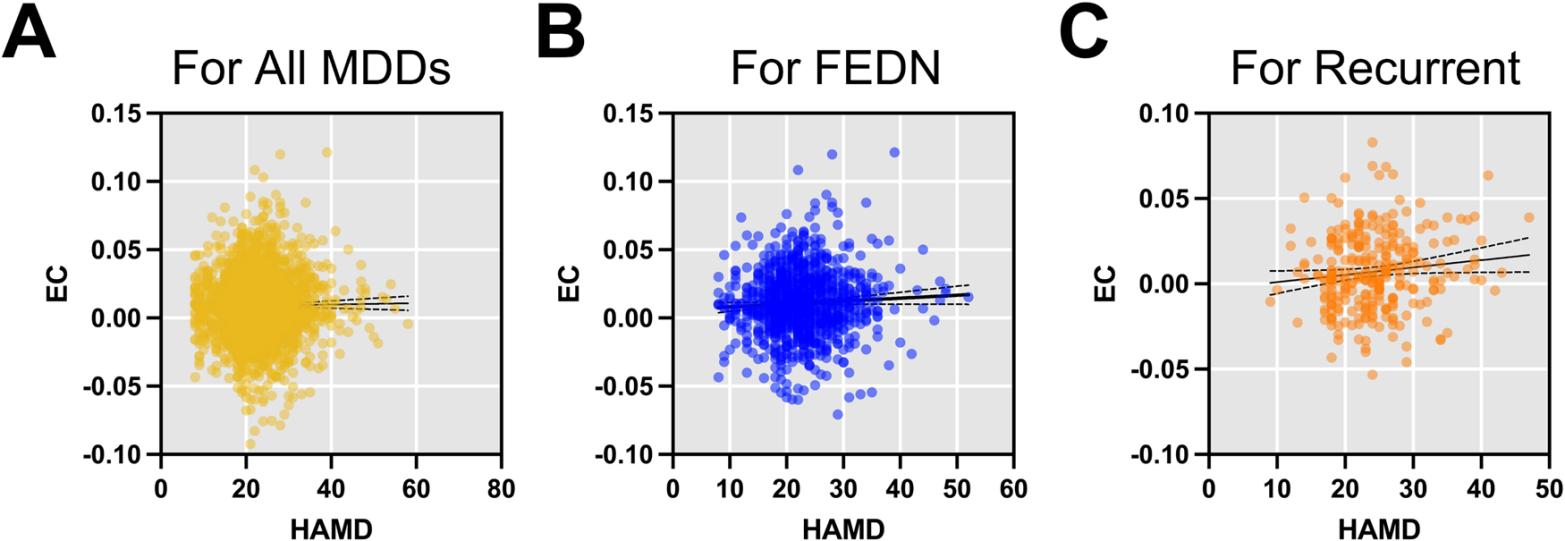
Association of EC from mPFC to PCC with symptom severity (HAMD). **A** All MDDs, **B** FEDN MDDs and **C** recurrent MDDs.

In this study, we used an unprecedentedly large sample of 4133 (2142 MDD patients and 1991 NCs) to investigate abnormal effective connectivity (EC) patterns within DMN and the validation of these patterns as potential biomarkers for predicting responses to both pharmacological and rTMS treatments was carried out using four datasets. Finally, in this study, we found a new neuroimage biomarker (EC from the mPFC to the PCC within DMN) and demonstrate that (1) significantly reduced EC from mPFC to PCC was observed in recurrent MDD patients compared with NCs and FEDN MDD patients (Fig. 1D, E); (2) reduced EC from mPFC to PCC in recurrent MDD patients was associated with illness duration and medication effect (Fig. 2); (3) EC from mPFC to PCC is anticipated to be significantly reduced in patients with MDD who demonstrate improvement following pharmacological treatment (Fig. 3). (4) EC from mPFC to PCC is associated with antidepressant treatment improvement rather core depressive symptoms including suicide, anhedonia, or emotional blunting (Figs. 4 and 5); (5) EC from mPFC to PCC could reliably discriminate between patients who responded positively to antidepressant treatment and those who did not (Fig. 6); (6) EC from mPFC to PCC could effectively predict therapeutic outcomes following antidepressant treatment (Fig. 7). This new biomarker provides a way forward in treatment evaluation, relapse prediction and understanding the pathogenesis of MDD.

## Methods

### Participants

This study included four data sets for major depression (mddrest, SAINT, Xijing_KG and Xijing_QG). All participants provided written informed consent at their local institution. Further study details are provided in the online supplement.

The mddrest data set collected by the REST-meta-MDD project^7^ from the Depression Imaging REsearch ConsorTium (DIRECT)^16^ in 2017, is currently the largest MDD dataset. Resting-state fMRI data from this data set were shared from twenty-five study cohorts in China. In this study, we used a sample of (*n* = 4133, 2142 MDD/1991 NCs) from both the first and second releases.

The SAINT dataset was approved by the Ethics Committee of the First Affiliated Hospital, Fourth Military Medical University (KY20202066-F-1) on October 21, 2020, and was registered with clinicaltrial.gov (identifier: NCT 04653337). All participants were recruited from the Department of Psychiatry at the First Affiliated Hospital, Air Force Military University, from January 2021 to October 2021. Thirty-two subjects received the Stanford Accelerated Intelligent Neuromodulation Therapy (SAINT)^17^. All treatments were delivered with a Black Dolphin Navigation Robot system (SmarPhin S-50, Solide Brain Control Medical Technology Co., Ltd, Xi’an, China). In a precise experimental protocol, three consecutive sessions of intermittent theta-burst stimulation (iTBS) were administered at 90% of the resting motor threshold (RMT) within a span of 9 min and 52 s. The subjects underwent a total of ten iTBS sessions, comprising 18,000 pulses, with a 50-min interval between each session. This daily regimen was sustained for five consecutive days, resulting in each patient receiving a cumulative total of 90,000 pulses throughout the entire treatment duration. According to exclusion and inclusion criteria, twenty-six treatment-resistant patients with major depression were enrolled in this study. Suicidal ideation severity was measured using the Chinese Version of the Beck Scale for Suicide Ideation (BSI-CV).

The Xijing_KG dataset was collected through Randomized Clinical Trials (RCTs). The dataset was approved by the Ethics Committee of the First Affiliated Hospital, Fourth Military Medical University (KY20222165-F-1) on May 24, 2023, and was registered with clinicaltrial.gov (identifier: NCT 05544071). All patients were recruited from the Psychosomatic Department of Xijing Hospital between March 2023 and April 2024. Inclusion criteria included (1) Diagnosis of MDD according to the criteria outlined in the Diagnostic and Statistical Manual of Mental Disorders, Fifth Edition (DSM-V), and currently experiencing an episode; (2) A total score greater than 17 on the Hamilton Depression Rating Scale (HAMD-17) at screening and baseline; (3) A total score greater than 20 on the Snaith-Hamilton Pleasure Scale (SHAPS) at screening and baseline; (4) No use of any antidepressant medications for at least 2 weeks prior to screening. Exclusion criteria included (1) Substance use disorders, excluding nicotine and caffeine; (2) Current diagnosis of any psychiatric disorders other than MDD, Generalized Anxiety Disorder, Social Anxiety Disorder, Panic Disorder, Agoraphobia, or Specific Phobias; (3) History of schizophrenia or schizoaffective disorder, or current or past depressive episodes with psychotic symptoms; (4) Other psychiatric disorders with depressive symptoms as the primary manifestation, personality disorders, or intellectual disabilities; (5) Significant clinical abnormalities identified during screening that may affect participation in the study or compromise safety, or that could interfere with the interpretation of study results; (6) Any history deemed by the investigator to pose a risk to the subject or interfere with the interpretation of study results; (7) Participation in other drug clinical trials or device clinical trials within 1 month prior to enrollment; (8) Presence of metal or electronic devices in the head or skull; (9) History of epilepsy; (10) History of cardiovascular disease or cardiovascular events; (11) History of obsessive-compulsive disorder; (12) History of autism spectrum disorder; (13) Prior exposure to repetitive transcranial magnetic stimulation (rTMS); (14) Any other conditions deemed by the investigator as unsuitable for participation in the study.

Patients in Xijing_KG dataset enrolled in the study will receive a selective serotonin reuptake inhibitor (SSRI), sertraline hydrochloride, at a daily dose of 100–150 mg. During the initial treatment phase (days 1–3), patients will receive 25 mg/day, with an increase to 50 mg/day on days 4–7. In the absence of dose-limiting adverse events, the dosage will be titrated to 100 mg/day during the second week and subsequently adjusted within the range of 100–150 mg/day to achieve optimal clinical response. Thereafter, the dosage will be maintained as consistently as possible. The medication is administered once daily, at a fixed time in the morning, either on an empty stomach or following a meal. Clinical evaluations will be conducted at baseline, immediately following the final session of repetitive transcranial magnetic stimulation (rTMS), and at 15 and 30 days post-rTMS. Anhedonia severity in patients with major depressive disorder (MDD) will be assessed using the Snaith-Hamilton Pleasure Scale (SHAPS) and the Chinese Version of the Temporal Experience of Pleasure Scale (CV-TEPS). Changes in depressive symptoms will be evaluated using the Montgomery-Asberg Depression Rating Scale (MADRS) and the 17-item Hamilton Depression Rating Scale (HAMD-17). Suicidal ideation severity will be measured using the Chinese Version of the Beck Scale for Suicide Ideation (BSI-CV). Additionally, MRI scans will be performed at baseline and on the 15th day after treatment.

The Xijing_QG dataset was collected through Randomized Clinical Trials (RCTs). The dataset was approved by the Ethics Committee of the First Affiliated Hospital, Fourth Military Medical University (XJLL-KY20222111) on May 24, 2023, and was registered with clinicaltrial.gov (identifier: NCT 05577481). Patients were recruited from the Department of Psychosomatic Medicine, Xijing Hospital, between March 2023 and January 2024. Inclusion Criteria included (1) Adults aged 18–60 years, right-handed; (2) Diagnosis of MDD based on DSM-V criteria; (3) A total score >17 on the 17-item Hamilton Depression Rating Scale (HAMD-17); (4) A score ≥6 on the Chinese Version of the Beck Scale for Suicide Ideation (BSI-CV); (5) Normal findings in physical examination, medical history, vital signs, and routine laboratory tests, including blood, urine, and stool analyzes, liver and kidney function, electrolytes, electrocardiogram, and electroencephalogram; (6) Informed of the safety profile of repetitive transcranial magnetic stimulation (rTMS), expressed willingness to comply with the treatment regimen, and provided signed informed consent. Exclusion Criteria included (1) Age <18 or >60 years; (2) History of severe physical illness or depression secondary to psychoactive substances or non-dependent substances; (3) Presence of metallic or electronic implants, such as intracranial metallic objects, cochlear implants, pacemakers, stents, or other metallic foreign bodies; (4) Risk of seizures, including a history of brain disease, head trauma, alcohol abuse, abnormal electroencephalogram findings, MRI evidence of structural brain abnormalities, or a family history of epilepsy; (5) Requiring immediate intervention due to suicidal behavior or severe self-injury; (6) Presence of psychotic symptoms requiring the use of antipsychotic medications; (7) History of electroconvulsive therapy (ECT) within 2 months prior to enrollment; (8) Pregnant, breastfeeding, or planning to conceive during the study period. Participants were required to have received at least 6 weeks of treatment with SSRIs or SNRIs prior to enrollment. During the study, the type of antidepressant was maintained, and the dosage was kept as close as possible to the pre-enrollment regimen. Concomitant use of medications for somatic diseases was permitted, provided the types and doses of these medications remained unchanged throughout the study. The treatment was administered continuously for 15 days, with clinical scales and cognitive function assessments conducted at baseline, day 7, and day 15 (end of treatment) following treatment. Emotional blunting in patients with MDD was assessed using the Oxford Depression Questionnaire (ODQ). Depressive symptom changes were measured using the HAMD-17. MRI scans were performed at baseline and at the conclusion of the 15-day treatment period.

### fMRI Preprocessing

Standard preprocessing of the mddrest was done at each site using the Data Processing Assistant for Resting-State fMRI (DPARSF, http://rfmri.org/DPARSF). Functional data of SAINT were preprocessed using the Graph Theoretical Network Analysis (GRETNA, https://www.nitrc.org/projects/gretna). Specifically, the initial ten volumes of each subject were discarded to account for potential instability in the signal. Slice-timing was performed to correct for temporal differences between slices. Then, realignment was conducted to correct for head motion during the acquisition by applying translation and rotation corrections to the fMRI images at different time points. The high-resolution T1-weighted images were co-registered with the functional images, and a segmentation process was performed to classify the brain into gray matter, white matter, and cerebrospinal fluid (CSF) compartments. The resulting deformation parameters from the T1-weighted images to the Montreal Neurological Institute (MNI) template was utilized to normalize the resting-state fMRI data to a standard space. Additionally, a Gaussian filter with a half maximum width of 6 mm was used to smooth the functional images. Each participant’s time series was band-pass filtered in the range of 0.01-0.1HZ. Finally, we regressed out the effects of head motion, white matter and CSF signals.

### Effective connectivity analysis

After preprocessing, we extracted time series for Dosenbach 160 functional regions of interest (ROIs)^18^. We then defined 33 DMN ROIs as those overlapping with the DMN delineated by Yeo et al.^19^ and calculated effective connectivity within DMN ROIs using granger causality analysis (GCA)^20^. In accordance with prior research findings^21,22^, we have delineated the Default Mode Network (DMN) into four principal components (Fig. 1A). These encompass 12 ROIs situated in the medial prefrontal cortex (mPFC), 11 ROIs located in the posterior cingulate cortex (PCC), 6 ROIs in the left parietal cortex (LPC), and 4 ROIs in the right parietal cortex (RPC). The analysis was performed using the REST-GCA toolbox (https://rfmri.org/REST-GCA), the order is set to 1 according to the previous study^23^. Prior to performing Granger Causality Analysis (GCA), the stationarity of each regional time series was assessed using the Augmented Dickey-Fuller (ADF) test. Time series that failed to meet the stationarity requirement (*p* < 0.05) were considered non-stationary. These non-stationary series were then differenced (first-order differentiation) iteratively until stationarity was achieved. All subsequent GCA analyzes were conducted exclusively on the preprocessed, stationary time series. Mean effective connectivity (EC) from mPFC to PCC (averaged across 12 × 11 = 132 connections) was computed (Fig. 1A). Granger causality analysis was executed using MATLAB scripts.

### Harmonization of site differences and covariates

To address site-specific artifacts and confounding effects, we employed the ComBat harmonization method^24,25^ to control for site differences and covariates in DMN EC. This approach enabled us to maintain biological variability while minimizing site-related variation. For each EC value, Combat model can be formulated as follows:

1 $$y={const}+{X}^{T}\beta +\gamma +\delta \varepsilon ,$$

in which ${const}$ represents the constant term; ${X}^{T}$ is a design matrix for the covariates of interest (age, sex, head motion); $\beta$ is the vector of coefficients associated with $X$; $\gamma$ represents additive site effects (location parameter), while $\delta$ denotes multiplicative site effects (scale parameter).

Accordingly, the harmonized EC values were defined as:

2 $${y}^{{Combat}}=\frac{y-\widehat{const}-{X}^{T}\hat{\beta }-\hat{\gamma }}{\hat{\delta }}+\widehat{const}+{X}^{T}\hat{\beta },$$

where $\hat{\delta }$ and $\hat{\gamma }$ represent the empirical Bayesian estimates of $\delta$ and $\gamma$, respectively.

### Group-level analysis

Linear mixed models (LMMs)^26^ were employed for between-group inference regarding EC from mPFC to PCC. This approach effectively controlled for confounding variables, including diagnosis, age, sex, education, head motion effects, and site-specific variations. The model was implemented using MATLAB’s fitlme command (https://www.mathworks.com/help/stats/fitlme.html), formulated as *y* ∼1 + Diagnosis + Age + Sex + Education + Motion + (1 | Site) + (Diagnosis | Site). This model provided *t* and *p* values for the fixed effect of Diagnosis. Cohen’s d effect size was subsequently computed as $d=\,\frac{T({n}_{1}+{n}_{2})}{\sqrt{{df}}\sqrt{{n}_{1}{n}_{2}}}$^27^.

In addition, we utilized LMM to compare 2142 MDD patients with 1991 NCs. Considering that several sites reported whether patients with MDD were in their first episode or recurrent, we compared 828 first-episode drug-naïve (FEDN) MDD patients with 812 corresponding normal controls (NCs) from nine sites and 310 recurrent MDD patients with 928 corresponding NCs from ten sites. Furthermore, we conducted a comparison between 516 FEDN MDD patients and 281 recurrent MDD patients from nine sites. In this particular analysis, the LMM model incorporated the replacement of the diagnosis variable with the FEDN or recurrent status to investigate specific distinctions between these subgroups. Due to the skewed distribution of illness duration, with the majority of cases being brief, we opted to contrast the terciles with the longest and shortest illness durations instead of using Diagnosis in the LMM. Additionally, to assess the impact of medication, we substituted Diagnosis with medication status (on/off, assessed at the time of the scan) in the LMM model. Finally, we used paired *t* test to compare the changes in EC between pre-treatment and post-treatment MDD patients.

### Prediction of antidepressant response using EC form mPFC to PCC

Support Vector Machine (SVM)^28^ classifiers were employed to predict the response to antidepressant treatment using EC from mPFC to PCC. In other words, the aim was to distinguish between patients who responded positively to antidepressant treatment and those who did not. In this study, we used a total of 315 MDD patients including 213 patients who exhibited a positive response to antidepressant treatment (Medication: 173 responders; rTMS: 40 responders) and 102 patients who showed no response to antidepressant treatment (Medication: 56 non-responders; rTMS: 46 non-responders). Specifically, 161 MDD patients from mddrest dataset (Medication: 145 responders versus 16 non-responders), 26 MDD patients from SAINT dataset (rTMS: 20 responders versus 6 non-responders), 72 MDD patients from Xijing_KG dataset (Medication: 9 responders versus 24 non-responders; rTMS: 12 responders versus 27 non-responders) and 40 MDD patients from Xijing_QG dataset (Medication: 3 responders versus 16 non-responders; rTMS: 8 responders versus 13 non-responders). In the context of the EC from mPFC to PCC, comprising $12\times 11=132$ connections, a meticulous approach was adopted to mitigate overfitting to noise in the data and prevent inflation of prediction performance. Thus, we employed the recursive feature elimination method^29^ to identify salient connections by recursively eliminating the least important ones based on model performance. This meticulous process yielded a selection of 38 connections for further analysis. Additionally, we applied regularization techniques (L1 regularization), during the feature selection process. Subsequently, the 38 identified connections were employed to train a Support Vector Machine (SVM) model utilizing the scikit-learn machine learning library^30^. In addition, we used the grid search function to generate candidate hyper-parameters, spanning from ${10}^{-5}$ to ${10}^{5}$ times the default values of C. Then C was set to 5. In addition, we utilized Support Vector Machine (SVM) with the ‘class_weight=balanced’ parameter, which adjusts the model to account for class imbalances during training. Finally, we implemented five-fold cross validation to assess performance and accuracy, area under the curve (AUC), sensitivity, specificity, *f*_1_-score, Matthews Correlation Coefficient (MCC)^31^ were used as evaluating indicators. To further ensure the reliability of the results, the entire feature selection and model training process was iterated 100 times, with the average performance serving as the final evaluation metric. To assess the statistical significance of the machine learning-based treatment prediction results, a nonparametric permutation test was conducted. Specifically, the treatment response labels were randomly shuffled across participants 1000 times, followed by the repeated application of fivefold cross-validation to generate a distribution of classification accuracies. The *p*-value was calculated as the proportion of the cross-validated accuracies from the permuted data that exceeded those obtained without permutation.

### Prediction of relative symptom improvement for rTMS treatment using EC from mPFC to PCC

We developed an end-to-end machine-learning algorithm for predicting the medication and rTMS treatment outcome using EC from mPFC to PCC features. Specifically, support vector regression (SVR) model was used to predict clinical improvements after medication or rTMS treatment for 138 patients with MDD (Medication: 52 participants; rTMS: 86 participants). Clinical improvements were quantified by subtracting the post-treatment scores from the pre-treatment scores on the 17-item Hamilton Depression Rating Scale (HAMD-17)^32^. Recursive feature elimination method was used to reduce the dimension of mPFC->PCC EC features and 38 salient connections were retained according to performance of the model. In addition, we used the grid search function to generate candidate hyper-parameters, spanning from ${10}^{-5}$ to ${10}^{5}$ times the default values of C. Then C was set to 9. The performance of five-out cross validation was quantified using the Mean Absolute Error (mae), Root Mean Squared Error (rmse) and Pearson’s correlation coefficient.

## Results

### Participant characteristics

In this study, 4133 participants in the mddrest dataset, 26 MDD patients in the SAINT dataset, 72 MDD patients in the Xijing_KG dataset and 40 MDD patients in the Xijing_QG were enrolled. Tables 1–4 demonstrate the demographic and clinical characteristics of the participants.

**Table 1:** Characteristics of participants in REST-meta-MDD cohort

Variable	MDD (*N* = 2930)		NC (*N* = 2445)		Analysis	
	*N*	%	*N*	%	χ^2^	*p*
Male	1062	36.25	1031	42.17	19.66	<0.0001
Currently employed	2142	73.11	1991	81.43		
	Mean	SD	Mean	SD	*t*	*p*
Age (years)	35.41	11.81	34.04	12.89	3.56	0.0004
Years of education	12.15	3.68	13.74	3.51	−14.23	<0.0001

*MDD* major depressive disorder, *NC* normal control.

**Table 2:** Characteristics of participants in SAINT cohort

Variable	Pre-treatment MDD (*N* = 32)		Post-treatment MDD (*N* = 32)		Analysis	
	*N*	%	*N*	%		
Male	6	19	6	19		
Currently employed	26	81	26	81		
	Mean	SD	Mean	SD	*t*	*p*
Age (years)	27.54	11.02	27.54	11.02	NA	NA
Years of education	12.81	2.83	12.81	2.83	NA	NA
17-item HAMD score	27.69	4.05	5.92	4.37	18.83	<0.0001
MADRS score	36.19	4.46	8.92	5.45	19.73	<0.0001
BSI-CV score	17.31	7.39	3.73	4.86	10.87	<0.0001

*HAMD* Hamilton Depression Rating Scale, *MADRS* Montgomery-Asberg Depression Rating Scale; *BSI-CV* Beck Scale for Suicidal ideation-Chinese Version.

**Table 3:** Characteristics of participants in Xijing_KG cohort

Variable	Pre-treatment MDD (*N* = 75)		Post-treatment MDD (*N* = 75)		Analysis	
	*N*	%	*N*	%		
Male	23	31	23	31		
Active rTMS	39	54	39	54		
Currently employed	72	96	72	96		
	Mean	SD	Mean	SD	*t*	*p*
Age (years)	14.83	1.22	14.83	1.22	N/A	N/A
SHAPS	37.19	5.65	36.19	6.99	0.94	0.35
17-item HAMD score	21.78	5.51	14.61	7.81	6.32	<0.0001

*HAMD* Hamilton Depression Rating Scale, *SHAPS* Snaith Hamilton Pleasure Scale.

**Table 4:** Characteristics of participants in Xijing_QG cohort

Variable	Pre-treatment MDD (*N* = 49)		Post-treatment MDD (*N* = 40)		Analysis	
	*N*	%	*N*	%		
Male	11	28	11	28		
Active rTMS	21	53	21	53		
Currently employed	40	82	40	82		
	Mean	SD	Mean	SD	*t*	*p*
Age (years)	36.48	12.29	36.48	12.29	N/A	N/A
ODQ	94.15	14.12	75.55	24.96	4.05	<0.0001
17-item HAMD score	16.15	5.04	10.08	6.07	4.81	<0.0001

*HAMD* Hamilton Depression Rating Scale, *ODQ* Oxford Depression Questionnaire.

### Reduced EC from mPFC to PCC within DMN in recurrent MDD patients

Mean EC from mPFC to PCC was compared with 2930 MDD patients and 2445 NCs, but no significant change (*t* = 0.051, *p* = 0.960, *d* = 0.0015; Fig. 1B) was observed. On subgroup analyzes, no significant change in FEDN MDD patients compared with NCs (*t* = 0.371, *p* = 0.711, *d* = 0.0063; Fig. 1C), while reduced EC from mPFC to PCC was observed in recurrent MDD patients compared with NCs (*t* = −2.969, *p* = 0.003, *d* = −0.1416; Fig. 1D). In addition, recurrent MDD patients showed significantly lower EC than FEDN MDD patients (*t* = −2.248, *p* = 0.025, *d* = −0.1690; Fig. 1D).

### DMN connectivity was associated with illness duration and medication status effect

The reduced EC observed from mPFC to PCC in recurrent MDD patients, as opposed to its absence in FEDN MDD patients, may result from the influence of illness duration or medication history. Significantly decreased EC from mPFC to PCC (*t* = −2.295, *p* = 0.022, *d* = −0.1809; Fig. 2A) was observed in the tercile with longest illness duration (≥24 mo, 1199 MDD patients from 29 sites) compared with the tercile with shortest illness duration (≤3 mo, 301 MDD patients from 15 sites). To reduce the impact of medication, we further examined the effect of illness duration in FEDN MDD patients. The tercile with longest illness duration (≥24 mo, 478 MDD patients from 23 sites) showed lower EC (*t* = −1.970, *p* = 0.049, *d* = −0.1998; Fig. 2B) than the tercile with shortest illness duration (≤3 mo, 272 MDD patients from 15 sites). Moreover, we conducted additional examinations to scrutinize the impact of medication. In particular, we compared individuals with FEDN MDDs (534 MDDs from 15 sites) with first-episode MDDs on medication (700 MDDs from 18 sites) and observed a significantly enhanced EC mPFC to PCC (*t* = 2.221, *p* = 0.027, *d* = 0.1434; Fig. 2C).

### Antidepressant treatment effect

Considering the association between medication status and EC from mPFC to PCC, we further investigated the changes of EC following the antidepressant treatment including medication treatment and rTMS treatment on three external prospective treatment datasets. To gain a more accurate understanding of the mechanisms, we divided the dataset based on treatment response. Here, responders are identified as individuals exhibiting a reduction of 50% or greater on the HAMD-17 scale following the specific antidepressant treatment (positive response), while non-responders are those who do not meet this threshold (negative response). Significantly enhanced EC from mPFC to PCC (*t* = 2.128, *p* = 0.044, *d* = 0.9425; Fig. 3A) was observed in the pre-treatment patients with MDD compared with post-treatment patients with MDD, when patients with MDD showed positive response following medication treatment. Additionally, we further tested an interaction between time and responder to more directly assess whether EC changes are specific to responders. Specifically, we employed a two-sample *t*-test to compare the change in EC from mPFC to PCC, calculated by subtracting pre-treatment values from post-treatment values, between responders and non-responders. This analysis revealed no significant difference (*t* = −1.09), suggesting that the observed changes in EC are not significantly different between responders and non-responders.

### Association of DMN connectivity with depressive symptom

The investigation delving into the relationship between EC from mPFC to PCC and HAMD scores underwent rigorous testing on REST-meta-MDD dataset (2369 MDD patients). Surprisingly, the analysis did not unveil a statistically significant correlation (Pearson’s *r* = 0.0069, *p* = 0.7359). When assessing the impact of symptom severity in FEDN MDD patients (*n* = 1022), the correlation failed to attain statistical significance (Pearson’s *r* = 0.0496, *p* = 0.1134). Conversely, among patients with recurrent MDD (*n* = 283), a significant correlation emerged (Pearson’s *r* = 0.1180, *p* = 0.0473).

Furthermore, we further investigated the relationship between pre- and post-treatment DMN connectivity (EC from mPFC to PCC) and depressive symptoms (as measured by BCI-CV, ODQ, SHAPS, and HAMD), as well as the association between pre-treatment DMN connectivity and treatment improvement (Fig. 5). Note that BCI-CV assesses suicidal ideation, ODQ evaluates emotional blunting, SHAPS measures anhedonia, and HAMD evaluates the severity of depression. We found that DMN connectivity, both pre- and post-treatment, was unrelated to depressive symptoms. However, as treatment progressed, pre-treatment DMN connectivity was able to predict changes in SHAPS (*r* = −0.31, *p* = 0.008) and ODQ scores (*r* = 0.34, *p* = 0.036).

### DMN Connectivity characterizes medication response

We further used DMN EC and EC from mPFC to PCC for predicting the response to all antidepressant treatment (213 responders versus 102 non-responders), medication treatment (173 responders versus 56 non-responders) and rTMS treatment (40 responders versus 46 non-responders). Briefly, we first trained SVM classifiers during the fivefold cross-validation using the EC from mPFC to PCC features as input. To ensure result stability, the process was iterated 100 times, with the average outcome serving as the ultimate performance metric for the model. The classifier achieved an accuracy of 78.6% (AUC, 76.5%; sensitivity, 81.2%; specificity, 71.8%; *f*_1_, 84.8%; MCC, 49.3%; permutation test-validated using 1000 permutations, *p* < 0.001) for the all treatment, an accuracy of 87.8% (AUC, 86.9%; sensitivity, 88.5%; specificity, 85.2%; *f*_1_, 92.2%; MCC, 65.0%; permutation test-validated using 1000 permutations, *p* < 0.001) for the medication treatment and an accuracy of 83.8% (AUC, 84.3%; sensitivity, 85.3%; specificity, 83.3%; *f*_1_, 81.3%; MCC, 68.0%; permutation test-validated using 1000 permutations, *p* < 0.001) for the rTMS treatment. Figure 6 shows sensitivity-specificity curves based on model predictions using EC from mPFC to PCC. Confusion Matrices and calibration plots are provided in Supplementary Fig. S4.

Furthermore, we further compared our proposed biomarker with several established biomarkers, including: (1) whole-brain functional connectivity (FC), (2) default mode network (DMN) FC, (3) medial prefrontal cortex - posterior cingulate cortex (mPFC-PCC) FC, (4) graph-theoretical measures such as betweenness, degree, clustering coefficient, efficiency, and local efficiency across five network nodes, and (5) clinical predictors including age, sex, and pre-treatment Hamilton Depression Rating Scale (HAMD) scores. To ensure robustness, we employed a Support Vector Machine (SVM) for model training across all biomarkers. The comparison results, summarized in Table 5, demonstrate that the effective connectivity (EC) from the mPFC to the PCC yielded the highest classification accuracy, emphasizing the superior performance of our biomarker and its promising potential for clinical application.

**Table 5:** Five-fold cross-validation performance across different biomarkers in antidepressant treatment (213 responders vs. 102 non-responders)

Biomarkers	ACC	AUC	Sensitivity	Specificity	F1	MCC
FC (Whole Brain)	0.617	0.585	0.503	0.668	0.401	0.152
FC (DMN)	0.610	0.592	0.494	0.689	0.470	0.178
FC (mPFC-PCC)	0.590	0.565	0.465	0.665	0.421	0.123
Graph-theoretical measures	0.496	0.467	0.334	0.600	0.323	−0.063
Clinical predictors	0.630	0.610	0.565	0.655	0.376	0.165
**Ours**	**0.786**	**0.765**	**0.812**	**0.718**	**0.848**	**0.493**

### Prediction of relative symptom improvement for rTMS using DMN connectivity

In addition to forecasting responses to medication treatment, we further employed EC from mPFC to PCC for predicting the therapeutic outcomes associated with medication and rTMS. We built prediction model using support vector regression method and model performance was tested using five-fold cross-validation (Fig. 7). The findings revealed a significant predictive capability of EC from mPFC to PCC for the observed changes in treatment scores during fivefold cross-validation (All treatment: Fig. 5A, Pearson’s *r* = 0.56, mae = 4.84, rmse = 6.36, *p* < 0.0001; Medication: Fig. 5B, Pearson’s *r* = 0.75, mae = 2.72, rmse = 4.24, *p* < 0.0001; rTMS: Fig. 5C, Pearson’s *r* = 0.72, mae = 4.07, rmse = 5.53, *p* < 0.0001).

## Discussion

In this study, employing an exceptionally large sample size (*n* = 4133), we identified a substantial reduction in top-down EC within the DMN, specifically from mPFC to PCC, in patients with recurrent MDD compared to both NCs and those with FEDN MDDs. Additionally, the diminished EC from mPFC to PCC in recurrent MDD patients showed associations with being scanned while on antidepressant medication and the duration of illness. Importantly, EC from mPFC to PCC demonstrated a remarkable capacity to predict treatment outcomes for both antidepressant medication and rTMS treatment.

Generally, the DMN is frequently partitioned into an anterior sub-network, which is focused on the mPFC, and a posterior sub-network, centered around the PCC^33^. Along the cortical midline, mPFC and PCC demonstrated robust functional coherence with both sub-networks, suggesting a potential role as functional hubs facilitating information transfer between sub-networks^34^. EC stands apart from conventional functional connectivity as it goes beyond simply computing the correlation among time courses of interacting regions. Instead, it possesses the capability to infer causal influences from one region to another, illustrating the directional flow of signals, including top-down and bottom-up processes, within a brain network. Significantly reduced effective connection from mPFC to PCC observed in the recurrent MDD group may imply a diminished flow of information from the anterior sub-network to the posterior sub-network within the DMN. Due to both sub-networks contributing to specific processes associated with self-generated thought^34^, this top-down regulation within DMN could be linked to the predominant clinical symptom of rumination in major depression^5,6^. Furthermore, the anatomical connection between the PCC and mPFC through the cingulum bundle in brain structure^35^ prompts the inquiry into whether structural abnormalities are present in patients with recurrent MDD compared to NCs, necessitating further investigation.

Significantly reduced EC from mPFC to PCC in recurrent MDD was associated with illness duration and antidepressant medication treatment. Specifically, we verified that the tercile with the longest illness duration (≥24 months) exhibited lower EC from mPFC to PCC compared to the tercile with the shortest illness duration (≤3 months), and individuals with FEDN MDD who were scanned while on medication displayed decreased EC from mPFC to PCC compared with those without medication. These results are consistent with a previous study, where the EC from the right parietal cortex (RPC) to the PCC within DMN exhibited a significant decrease in patients with post-treatment MDD compared with NCs, after an eight-week treatment period^36^. Our identification of a medication-induced decrease in EC from mPFC to PCC implies that antidepressant medications could mitigate depressive symptoms by reducing the EC from mPFC to PCC. To minimize the influence of medication, we conducted additional analyzes to explore the impact of illness duration in FEDN MDD patients. Among FEDN MDD patients, those in the tercile with the longest illness duration (≥24 months) displayed decreased EC from mPFC to PCC in comparison to those in the tercile with the shortest illness duration (≤3 months). This result suggests that the prolonged impact of the illness may lead to a lower EC from mPFC to PCC. However, the identified medication effect and illness duration effect were observed in a retrospective cross-sectional sample, underscoring the need for validation through longitudinal designs integrating medication and disease follow-up.

Another significant finding of the present study is the capacity of EC from mPFC to PCC within DMN to reliably predict treatment responses to both medication and rTMS. Despite receiving optimal treatment for MDD, only 30% of patients achieve full recovery or remission. The remaining 70% either respond without achieving remission (around 20%) or show no response at all (50%)^37^, commonly termed as treatment-resistant depression (TRD). Individuals with Treatment-Resistant Depression (TRD) bear the highest direct and indirect medical costs among those with MDD^38^. Specifically, TRD individuals have twice the probability of hospitalization, incurring a cost exceeding six times the mean total cost for non-treatment-resistant depressed patients^39^. Hence, there is a pressing need for objective biomarkers to identify TRD and inform individualized treatment decisions. In this study, EC from mPFC to PCC reliably differentiated patients with positive responses to medication treatment from those who did not respond and predicted therapeutic outcomes following rTMS treatment among individuals with TRD. This suggests that the identified fMRI signature holds promise as a neuroimaging biomarker for guiding treatment choices in the context of antidepressant response. Furthermore, this might lead to avoidable morbidity and economic costs if individuals are transitioned to another intervention prematurely based on evidence indicating minimal expected benefit from antidepressant treatment using our fMRI signature.

Our findings contribute to the growing body of evidence surrounding the role of the default mode network (DMN) in brain function and its potential as a therapeutic target. While repetitive transcranial magnetic stimulation (rTMS) has been widely utilized in clinical practice, it has traditionally focused on the emotional network (sgACC) and cognitive control network (DLPFC). Despite the known pivotal role of the DMN in various cognitive and emotional processes, it has not yet been targeted for therapeutic intervention. Our study reveals a significant association between the DMN and treatment outcomes, providing strong evidence for the feasibility of DMN-targeted interventions. This novel approach could offer new avenues for therapeutic interventions, particularly for individuals with disorders involving DMN dysfunction. Moving forward, targeted rTMS stimulation within the DMN holds promise as a viable strategy for treating a range of neuropsychiatric conditions, warranting further investigation into its therapeutic potential.

The limitations of this study should be noted. First, our findings and biomarkers need validation in independent datasets. In the future, inclusion of UK Biobank MDD data and data from the ENIGMA-MDD consortium could be considered for analysis. Second, we only considered pharmacological treatment and rTMS, while other treatments with potentially distinct mechanisms of action, such as electroconvulsive therapy (ECT) or psychotherapy, should also be taken into consideration. Third, we employed fMRI signatures to distinguish patients exhibiting positive responses to medication treatment from non-responders, but we did not conduct a detailed investigation based on the specific types of medication. Finally, despite our efforts to harmonize these datasets, residual site-specific differences may persist, and some of these variations may not be fully accounted for or corrected by ComBat.

## Supplementary information

Supporting information

## Data Availability

Deidentified and anonymized data were contributed from studies approved by local Institutional Review Boards. All study subjects provided written informed consent at their local institution. Data of REST-meta-MDD I are available at: http://rfmri.org/REST-meta-MDD. Data of REST-meta-MDD II and SAINT used during the current study are available from the corresponding author upon reasonable request.
